# Whose responsibility? Part 1 of 2: A scale to assess how stakeholders apportion responsibilities for addressing the needs of persons with mental health problems

**DOI:** 10.1186/s13033-021-00510-x

**Published:** 2022-01-10

**Authors:** Srividya N. Iyer, Megan Pope, Aarati Taksal, Greeshma Mohan, Thara Rangaswamy, Heleen Loohuis, Jai Shah, Ridha Joober, Norbert Schmitz, Howard C. Margolese, Ramachandran Padmavati, Ashok Malla

**Affiliations:** 1 Department of Psychiatry, McGill University, Montreal, QC Canada; 2 Prevention and Early Intervention Program for Psychosis (PEPP-Montreal), Douglas Mental Health University Institute, Montreal, QC Canada; 3 Schizophrenia Research Foundation (SCARF), Chennai, India; 4 McGill University Student Services, McGill University, Montreal, QC Canada; 5 Department of Population-Based Medicine, Institute of Health Sciences, University Hospital Tübingen, Tübingen, Germany; 6 Prevention and Early Intervention Program for Psychosis (PEPP-MUHC), McGill University Health Centre, Montreal, Canada

**Keywords:** Responsibility, Mental health, Psychosis, Culture, Stakeholder participation, Assessment/measure, Government, Low-and middle-income countries, Family, Shared decision-making

## Abstract

**Background:**

Individuals with mental health problems have multiple, often inadequately met needs. Responsibility for meeting these needs frequently falls to patients, their families/caregivers, and governments. Little is known about stakeholders' views of who should be responsible for these needs and there are no measures to assess this construct. This study’s objectives were to present the newly designed Whose Responsibility Scale (WRS), which assesses how stakeholders apportion responsibility to persons with mental health problems, their families, and the government for addressing various needs of persons with mental health problems, and to report its psychometric properties.

**Methods:**

The 22-item WRS asks respondents to assign relative responsibility to the government versus persons with mental health problems, government versus families, and families versus persons with mental health problems for seven support needs. The items were modelled on a World Values Survey item comparing the government’s and people’s responsibility for ensuring that everyone is provided for. We administered English, Tamil, and French versions to 57 patients, 60 family members, and 27 clinicians at two early psychosis programs in Chennai, India, and Montreal, Canada, evaluating test–retest reliability, internal consistency, and ease of use. Internal consistency estimates were also calculated for confirmatory purposes with the larger samples from the main comparative study.

**Results:**

Test–retest reliability (intra-class correlation coefficients) generally ranged from excellent to fair across stakeholders (patients, families, and clinicians), settings (Montreal and Chennai), and languages (English, French, and Tamil). In the standardization and larger confirmatory samples, internal consistency estimates (Cronbach’s alphas) ranged from acceptable to excellent. The WRS scored average on ease of comprehension and completion. Scores were spread across the 1–10 range, suggesting that the scale captured variations in views on how responsibility for meeting needs should be distributed. On select items, scores at one end of the scale were never endorsed, but these reflected expected views about specific needs (e.g., Chennai patients never endorsed patients as being substantially more responsible for housing needs than families).

**Conclusions:**

The WRS is a promising measure for use across geo-cultural contexts to inform mental health policies, and to foster dialogue and accountability among stakeholders about roles and responsibilities. It can help researchers study stakeholders’ views about responsibilities, and how these shape and are shaped by sociocultural contexts and mental healthcare systems.

**Supplementary Information:**

The online version contains supplementary material available at 10.1186/s13033-021-00510-x.

## Background

Persons with mental health problems have multiple support needs which are often inadequately met [1, 2]. Their needs tend to pertain to areas such as housing, employment, education, treatment, and emotional and financial support [1, 2]. Responsibility for meeting these needs is generally assumed by a variety of stakeholders, particularly patients, their families or caregivers, and governments. While services and treatment providers also play a role in mental health needs provision, it falls often to patients and families to access services and to cover their costs fully or partially, depending on the nature of the healthcare system. Moreover, in all countries, governmental policy and funding decisions shape the larger context of needs provision, i.e., how mental health services are funded, organized, and provided. Despite this landscape, little is known about how various stakeholders think about responsibility for fulfilling these support needs, including how they believe such responsibility should be apportioned.

Globally, there is a great discrepancy between countries in terms of government support for treatment, housing, employment, education, and income for persons with mental disorders [3, 4]. Ninety-six percent of high-income countries (HICs) report availability of such support compared with only 14% of low- and middle-income countries (LMICs) [5]. Even within HIC and LMIC groups, there is wide variation in the amount of coverage provided by governments [6–8].

Historically, there has been relatively little user input into the structure, functioning, orientation or even the outcomes judged to be salient in mental health services. With the extensive endorsement of recovery-oriented frameworks of mental health services, particularly in HICs [9–11], patients have a greater say in defining their own recovery, and what role they themselves want to play in it.

Across contexts, families are also known to play critical roles in caregiving and supporting recovery, especially for serious mental illnesses such as psychosis [12–15]. In some LMICs, families have always contributed to addressing various needs of persons with mental health problems, given prevailing cultural norms [16, 17] and the paucity of formal mental health resources [18]. Cultural norms and ideologies related to autonomy [19], family piety [20] and notions of duty [21] also dictate when individuals assume responsibility for their health, and when family or government involvement is expected. Family caregivers' evolving expectations of their mentally ill relative over time [22] also may shift their views of their responsibilities as compared to the responsibilities of other stakeholders.

A better understanding of the extent to which various stakeholders perceive one another as being responsible for providing for the needs of persons with mental illnesses would help clarify how mental healthcare structures and sociocultural contexts shape and are shaped by expectations in this regard [23]. Perceived roles and responsibilities have considerable implications for mental health policies, funding decisions, and the design of mental health services [24], which are currently undergoing substantial reform globally through the growing early intervention and integrated youth mental health movements [25–27]. Furthermore, on a day-to-day basis, each stakeholder’s perception of their own and others’ roles and responsibilities is also likely to affect the amount and type of support they actually extend.

In the context of our longitudinal comparative study of 2-year prospective outcomes of first-episode psychosis in India and Canada [28, 29], focus groups were conducted with patients, families, and clinicians, revealing differences in perceived stakeholder responsibilities across the two settings and between stakeholder groups. These varying perspectives on responsibility may contribute to differential outcomes across sociocultural settings.

Furthering our knowledge obtained through these qualitative studies requires a quantitative measurement of stakeholders’ relative attributions of responsibility to governments, families, and patients for addressing various needs of individuals with mental health problems in different settings. Currently, there are no measures available to achieve this objective. Thus, our aims in this two-part report are to present a newly designed measure, the *Whose Responsibility Scale (WRS),* a 22-item instrument in which patients, family members, and clinicians assign relative responsibility to persons with mental health problems, families, and the government for various aspects of the treatment/recovery of persons with mental health problems (Part 1, current paper), and to discuss findings from our larger study utilizing the WRS to examine differences between stakeholders in Chennai, India and Montreal, Canada in their attributions of responsibility to persons with mental health problems, families, and the government (Part 2). In this paper, we describe the WRS and detail its development and psychometric properties.

## Methods

### Research context

As part of a larger cross-cultural investigation of first-episode psychosis [28, 29], this study was carried out with patients, families, and clinicians at two early intervention programs for youth with psychosis in Montreal, Canada and Chennai, India. Participants in Chennai were recruited from the first-episode psychosis clinic of the Schizophrenia Research Foundation (SCARF), a not-for-profit, non-governmental mental health organisation. In Montreal, participants were recruited from two nodes of McGill University’s publicly funded Prevention and Early Intervention Program for Psychosis (PEPP) [30]. The Chennai and Montreal programs are open to referrals from a wide variety of sources and offer a similar protocol of intensive, phase-specific, two-year psychosocial (assertive case management, family interventions, family psychoeducation, therapy, etc.) and medical (flexible use of low-dose antipsychotic medication) treatments provided by a multidisciplinary team of clinicians (case managers and psychiatrists) to persons with first-episode psychosis [29]. Persons treated with antipsychotic medication for more than 30 days or with IQ below 70, solely substance-induced or organic psychosis, pervasive developmental disorder, or epilepsy are excluded from each program. Concurrent substance use is not an exclusion criterion.

The study was approved by the relevant institutional ethics boards in Montreal and Chennai, and all participants gave informed consent. In the case of individuals younger than 18 years, participants provided assent and their parents/guardians provided consent.

### Participants

The standardisation sample comprised of 57 patients, 60 family members, and 27 clinicians across study sites (Table 1). Patients were included if they had a first episode of either affective or non-affective psychosis as determined by the Structured Clinical Interview for DSM IV-TR for Axis I Disorders (SCID-IV; [31]), were between 16–35 years old, and were fluent in English or French in Montreal, and English or Tamil in Chennai. The exclusion criteria were the same as that for the PEPP and SCARF programs. Family members were parents, spouses/partners, or siblings of patients with first-episode psychosis, and clinicians were case managers, psychiatrists, and other allied healthcare professionals (e.g., employment support specialists, psychologists, etc.) providing treatment at PEPP and SCARF.

**Table 1:** Sample characteristics

Variable		Montreal M (SD)/Na (%)	Chennai M (SD)/N (%)	Statistical Test (df)	p-value
Patient sample		N = 30	N = 26		
Age at entry (years)		23.75 (5.08)	26.23 (5.34)	t(54) = 1.775	**0.082**
Gender N (%)				χ^2^(2) = 0.045	0.832
Men		17 (56.7)	14 (53.8)		
Women		13 (43.3)	12 (46.2)		
Education (years)		12.16 (2.29)	12.46 (3.99)	t (38.593) = 0.332	0.742
Family sample		N = 29^b^	N = 28		
Age range in years	21–30	3 (10.3)	3 (10.7)	χ^2^(4) = 14.177	**0.007**
	31–40	1 (3.4)	11 (39.3)		
	41–50	5 (17.2)	2 (7.1)		
	51–60	16 (55.2)	12 (42.9)		
	61–70	4 (13.8)	0		
Gender	Men	5 (17.2)	13 (46.4)	χ^2^(1) = 5.617	**0.018**
	Women	24 (82.8)	15 (53.6)		
Education	Less than high school	1 (3.4)	1 (3.6)	χ^2^(5) = 18.966	**0.002**
	High school	7 (24.1)	21 (75)		
	College/vocational degree/ Diploma	8 (27.6)	1 (3.6)		
	Bachelor's degree	10 (34.5)	2 (7.1)		
	Master's degree	3 (10.3)	2 (7.1)		
	Doctoral degree	0	1 (3.6)		
Relationship with patient	Parent	23 (79.3)	14 (50)	χ^2^(3) = 9.908	**0.019**
	Spouse/ Partner	0	7 (25)		
	Sibling	4 (13.8)	6 (21.4)		
	Other	2 (6.9)	1 (3.6)		
Clinician sample		N = 12	N = 15		
Age range in years	21–30	1 (8.3)	11 (73.3)	χ^2^(3) = 14.288	**0.003**
	31–40	7 (58.3)	2 (13.3)		
	41–50	1 (8.3)	2 (13.3)		
	51–60	3 (25)	0		
Gender	Men	6 (50)	2 (13.3)	χ^2^(1) = 4.299	**0.038**
	Women	6 (50)	13 (86.7)		
Profession	Case Manager	8 (66.7)	15 (100)	χ^2^(2) = 5.87	0.053
	Physician/Psychiatrist	2 (16.7)	0		
	Other Allied Healthcare Professionals	2 (16.7)	0		

Bold indicates statistical significance

^a^Ns vary due to missing data

^b^Though number of family participants was 31 at Montreal, demographic data were available for only 29 of these participants

### Whose responsibility scale (WRS): development and description

The WRS was developed with multiple rounds of iterative discussion, feedback, and modifications involving clinician-scientists and mental health professionals at both sites with extensive experience in providing mental health care, including for persons with psychosis.

The WRS (Additional file 1) was modelled after an item that has been part of various rounds of the World Values Survey [32]. The item is rated on a 10-point scale, where 1 indicates complete agreement with the statement on the left pole of the scale (“The government should take more responsibility to ensure that everyone is provided for”), 10 indicates complete agreement with the statement on the right pole of the scale (“People should take more responsibility to provide for themselves”), and selection of any other number in between these poles reflects one’s relative weighting of the responsibility of each party. We included this item in the WRS, followed by a set of 21 similarly worded and structured items organized around seven needs of individuals with mental health problems: (1) general financial support, (2) housing support, (3) help with return to school/work, (4) help covering the costs of mental health services, (5) medication, (6) substance use treatment programs, and (7) mental health awareness-building and stigma reduction. These seven needs were selected based on previous literature [1, 33–39]; our clinical and program development experience; and focus group discussions on responsibilities of various stakeholders for supporting people with mental health problems, conducted with patients, families, and clinicians at PEPP and SCARF [40–42]. An initial version of the scale was also shared with three patients and two family members and their feedback was used to refine the scale and its instructions.

For each of these seven needs, a set of three items each rated on a 10-point scale are presented, with the first requiring respondents to contrast the role of the government with the role of persons with mental health problems, the second contrasting the role of the government with the role of families of persons with mental health problems, and the third contrasting the role of families with the role of persons with mental health problems (see Additional file 1). Respondents are asked to consider the attribution of responsibilities for supporting persons with mental health problems generally, and not with respect to their own specific case. In total, 22 items were included in the WRS.

The WRS was translated from English into Tamil and French and back-translated using standardized procedures recommended by the World Health Organization [43]. The WRS can be completed as a self-report or can be interviewer-administered.

### WRS scoring

The first item of the WRS (taken directly from the World Values Survey [32]) is a stand-alone item and does not contribute to the scoring of the WRS, insofar as it does not specifically tap into any of the seven need areas that subsequent questions are organized around. However, it was included in the scale as a useful indicator of respondents' general views of individual versus governmental responsibility for need provision, and scores on this item can be compared to scores on the more specific questions to determine whether respondents' general views of locus of responsibility differ from their views of locus of responsibility for specific areas of support. The remaining 21 items can be scored to arrive at three composite scores reflecting the extent of responsibility attributed to (1) governments versus persons with mental health problems, (2) governments versus families, and (3) families versus persons with mental health problems for addressing all needs taken together.

To arrive at these three composite scores, all items comparing government and patient responsibilities are summed to derive the first composite score; all items comparing government and family responsibilities are summed to derive the second composite score; and all items comparing patient and family responsibilities are summed to derive the third composite score. Alternatively, scores on each individual item can be summed and then averaged across participants to arrive at more easily interpretable scores between 1 and 10.

Depending on the research or policy objectives, one may in some cases need to estimate attributions of responsibility regarding specific needs. If so, similar composite scores can also be derived for each of the seven needs separately (e.g., if one is particularly interested in perceptions of responsibility for housing support, the three items corresponding to this need can be summed and averaged in the same way across participants).

### Procedure

To establish its psychometric properties, the WRS was administered as a self-report by trained research assistants to all participants (patients, families, and clinicians), in English or Tamil in Chennai and English or French in Montreal depending on participants’ linguistic preference, at two time points approximately two weeks apart in order to establish its test–retest reliability. Trained research staff were available to answer questions if needed and to administer a feedback questionnaire to a subset of the participants. We examined the internal consistency of the WRS using data obtained from the first administration of the measure. Internal consistency estimates were calculated for each set of seven items that were summed to derive responsibilities attributed to government versus persons with mental health problems, government versus families, and families versus persons with mental health problems. The internal consistency of the WRS was confirmed using the larger patient and family samples from the main comparative study (see Part 2 of this 2-part series).

We also examined the frequency distributions of WRS scores to see if respondents used all or part of the possible range (1–10) of item scores, by looking at whether there were scores between 1–3, 4–6, and 7–10 for each item.

To assess the scale's acceptability, we created a brief, easy-to-understand feedback questionnaire. This questionnaire includes two items that ask participants to rate the ease of comprehension and completion of the WRS on a 10-point Likert-type scale (1 = *very difficult* to 10 = *very easy*), and a third categorically-rated item (Was the WRS measure difficult to answer? – Yes/Somewhat/No). A subset of patients was approached to complete the feedback questionnaire (4–5 patients in each language, English and French in Montreal and English and Tamil in Chennai).

### Data analyses

Data were analysed using IBM SPSS version 22. Descriptive data regarding the sample characteristics were represented as means, standard deviations (SDs), and percentages. The samples from the two sites were compared using independent samples t-tests or chi square tests, using 0.05 as the significance level. Test–retest reliability for patient, family, and clinician groups were computed separately using intra-class correlation coefficient (ICC), with a two-way random effects model of variance and absolute agreement between scores at the two time points (ICC_2, 1_) and single measure estimates [44] with a 95% confidence interval. The ICCs were interpreted as recommended by Cicchetti [45]: “poor” (ICC < 0.40), "fair" (0.40–0.59), “good” (0.60–0.74) and "excellent" (ICC > 0.75). Disaggregated and combined reliability estimates were computed for Montreal and Chennai for the patient and family samples. Due to the smaller size of the clinician sample at each site, only combined reliability estimates were computed. Finally, test–retest reliabilities were assessed separately for the English, French, and Tamil versions of the WRS based on combined patient and family samples.

In the standardization sample, internal consistency was computed for patient, family, and clinician samples, aggregated and disaggregated across sites. Internal consistency reliabilities were also assessed separately for the English, French, and Tamil versions of the WRS based on combined patient and family samples.

Internal consistency estimates were then calculated for confirmatory purposes using the larger study samples at both sites (see details of larger study in Part 2 of this 2-part paper series). This analysis excluded those participants whose data was included in the smaller standardization sample. Estimates were computed separately by stakeholder (patient, family and clinician); site (Montreal, Chennai); and language (English and French in Montreal; English and Tamil in Chennai).

Often, reliability estimates based on internal consistency (Cronbach’s alphas) are interpreted as acceptable if > 0.70, good if > 0.80, and excellent if > 0.90 [46]. It has been argued that values below 0.60 are acceptable for exploratory research [47, 48] and that interpretation of such estimates be guided by how the scale was conceptualized rather than based on strict cut-offs [49, 50]. In our study, it was decided a priori that the measure would be considered internally consistent if the Cronbach's alpha estimates were at least 0.60. Higher internal consistency estimates were not consistently expected as it was theorized that ratings (e.g., for government versus persons with mental health problems) could reflect not only latent values about stakeholder responsibilities (e.g., a general belief that governments should take substantial responsibility for addressing key needs of persons with mental health problems), but also be influenced by the specific need area being rated (e.g., governments could be seen as more responsible for covering the costs of mental health services than the costs of substance use services).

## Results

### Sample description (Table 1)

Patients at both sites (n = 30 Montreal; n = 26 Chennai) were comparable in age (mid-twenties), years of education (12 years), and gender distribution (Table 1). The WRS was completed in French by 13 patients in Montreal and in Tamil by 18 patients in Chennai, with the remaining patients responding in English at both sites. Families at both sites (n = 31 Montreal; n = 28 Chennai) differed significantly on some demographic variables. More family members in Montreal were women, were in the 51–60 age group, had greater than high school education, and were parents of patients with FEP. In Chennai, gender distribution was comparable for male and female family members, a sizeable minority of family members were in the 31–40 age group, one quarter of family members were spouses/partners, and three quarters had high school education. The WRS was completed in French by 17 family members in Montreal, and in Tamil by 24 family members in Chennai. Clinicians in Chennai were all case managers and predominantly women (86%) in the 21–30 age group (73%), compared to Montreal, where two-thirds were case-managers, gender distribution was equal, and the majority were in the 31–40 age group. Description of the larger study samples can be found in Part 2 of this 2-part paper series.

### Test–retest reliability analysis (Table 2)

**Table 2:** Test–retest reliability based on ICC(2,1), with single measure estimates

Sample		Government-Patient ICC(2,1), (CI 95%) (N)^a^	Government-Family ICC(2,1), (CI 95%) (N)	Family-Patient ICC(2,1), (CI 95%) (N)
Patients	Total	0.709 (0.544, 0.821) (53)	0.566 (0.35, 0.724) (53)	0.569 (0.351, 0.728) (52)
	Montreal	0.741 (0.509, 0.873) (27)	0.602 (0.293, 0.796) (27)	0.44 (0.074, 0.702) (26)
	Chennai	0.651 (0.365, 0.826) (26)	0.483 (0.142, 0.726) (26)	0.736 (0.498, 0.872) (26)
Families	Total	0.705 (0.541,0.816) (57)	0.73 (0.581, 0.832) (27)	0.79 (0.665, 0.872) (55)
	Montreal	0.838 (0.646, 0.925) (28)	0.827 (0.641, 0.918) (28)	0.757 (0.526, 0.884) (26)
	Chennai	0.539 (0.228, 0.752) (29)	0.649 (0.376, 0.818) (29)	0.820 (0.653, 0.911) (29)
Clinicians	Total	0.883 (0.759, 0.945) (27)	0.924 (0.935, 0.966) (25)	0.768 (0.541, 0.891) (25)
Language	French (Patient = 12; Family = 17)	0.807 (0.632, 0.904) (29)	0.792 (0.606, 0.896) (29)	0.820 (0.637, 0.915) (26)
	English (Patient = 23; Family = 16)	0.819 (0.681, 0.901) (39)	0.707 (0.506, 0.835) (39)	0.576 (0.321, 0.753) (39)
	Tamil (Patient = 18; Family = 24)	0.455 (0.187, 0.662) (42)	0.541 (0.285, 0.725) (42)	0.738 (0.564, 0.849) (42)

^a^Ns vary due to missing data

All correlation coefficients were statistically significant (*p* < 0.05)

For the combined patient sample, the ICCs (2,1) were 0.71 (good), 0.57 (fair), and 0.57 (fair) for total responsibility ratings assigned to government versus persons with mental health problems, government versus families, and families versus persons with mental health problems, respectively. At each site, ICCs (2,1) were generally in the good range for the patient samples, with two exceptions where it was in the fair range.

For the combined family sample, the ICCs (2,1) were 0.71 (good), 0.73 (good), and 0.79 (excellent) for total responsibility ratings assigned to government versus persons with mental health problems, government versus families, and families versus persons with mental health problems, respectively. At each site, ICCs (2,1) were generally in the excellent range for the family samples, with two exceptions where it was in the good or fair range.

For the combined clinician sample, the ICCs (2,1) were 0.88 (excellent), 0.92 (excellent), and 0.77 (excellent) for total responsibility ratings assigned to government versus persons with mental health problems, government versus families, and families versus persons with mental health problems respectively.

Generally, we found excellent to good test–retest reliability for all language versions of the WRS. The exceptions were for responsibility assigned to persons with mental health problems versus families in the English version, and for responsibility assigned to the government versus persons with mental health problems and the government versus families in the Tamil version, where, in each case, the ICC (2,1) was only in the fair range.

A closer examination of raw scores across the two administrations of the WRS in Chennai indicated that the responses were in fact stable across time for the large majority of raters, with only three exceptions. Specifically, two patient raters attributed dramatically different levels of responsibility to governments versus families from Time 1 to Time 2, going from composite scores of 14 and 14, suggesting government was seen as relatively more responsible, to composite scores of 40 and 50, suggesting families were seen as relatively more responsible. Similarly, one family rater went from a composite score of 7 at Time 1 (suggesting government was seen as relatively more responsible versus persons with mental health problems) to a composite score of 61 at Time 2 (suggesting persons with mental health problems were seen as relatively more responsible). When the reliability estimates were re-calculated for Chennai after removing these three exceptions, the ICC values moved to the 0.688–0.979 (good–excellent) range (Additional file 2).

In summary, generally, responsibility ratings on the WRS tend to be stable over short time periods. Given that composite scores are based on seven items each, the dramatic change in composite scores between Times 1 and 2 in the case of the three exceptions suggests that raters' attributions of responsibility genuinely shifted over time from one stakeholder group (e.g., governments) to the other (e.g., patients), rather than erratically fluctuating over time.

### Internal consistency reliability analysis (Table 3)

**Table 3:** Internal consistency reliability estimates for patient, family and clinician samples (overall and by site)

Rater	Sample	N	No of items	Pair	Cronbach’s α
Patient	Total	56	7	Government-Patient	0.899
		56	7	Government-Family	0.878
		56	7	Family-Patient	0.85
	Montreal	30	7	Government-Patient	0.955
		30	7	Government-Family	0.966
		30	7	Family-Patient	0.901
	Chennai	26	7	Government-Patient	0.766
		26	7	Government-Family	0.628
		26	7	Family-Patient	0.744
Family	Total	59	7	Government-Patient	0.897
		59	7	Government-Family	0.918
		58	7	Family-Patient	0.848
	Montreal	31	7	Government-Patient	0.963
		31	7	Government-Family	0.934
		30	7	Family-Patient	0.924
	Chennai	28	7	Government-Patient	0.835
		28	7	Government-Family	0.923
		28	7	Family-Patient	0.817
Clinician	Total	25	7	Government-Patient	0.822
		25	7	Government-Family	0.793
		25	7	Family-Patient	0.643

All Cronbach’s α values were statistically significant (*p* < 0.05)

For the patient sample, Cronbach's alpha estimates were good for all three sets of items (government-persons with mental health problems, government-family, and family-persons with mental health problems) for the combined Montreal and Chennai samples; excellent for the Montreal sample; and acceptable for the Chennai sample. In the family sample, Cronbach’s alphas for the three sets were between good and excellent for the combined Montreal and Chennai samples, excellent for the Montreal sample, and good to excellent for the Chennai sample. In the clinician sample, Cronbach's alphas were acceptable for the family-persons with mental health problems and the government-family set of items, and good for the government-persons with mental health problems set of items. With one exception (which was acceptable), internal consistency estimates were in the good to excellent range for the three language groups (see Table 4).

**Table 4:** Internal consistency-based reliability estimates across language groups

Language	N	No of items	Pair	Cronbach’s α
English (Patient = 25; Family = 17)	42	7	Government-Patient	0.94
	42	7	Government-Family	0.895
	42	7	Family-Patient	0.892
French (Patient = 13; Family = 18)	31	7	Government-Patient	0.933
	31	7	Government-Family	0.94
	30	7	Family-Patient	0.889
Tamil (Patient = 18; Family = 24)	42	7	Government-Patient	0.808
	42	7	Government-Family	0.895
	42	7	Government-Patient	0.797

All Cronbach’s α values were statistically significant (*p* < 0.05)

Results from the larger sample confirmed the internal consistency of the WRS for all three stakeholder groups at each site; for the English and French versions at Montreal and the English and Tamil versions at Chennai. As indicated in Tables 5 and 6, Cronbach’s alphas generally ranged between excellent and good.

**Table 5:** Internal consistency for the larger study sample of patients and families

Rater	Sample	N	No of items	Pair	Cronbach’s α
Patient	Montreal	63	7	Government-Patient	0.933
		64	7	Government-Family	0.935
		64	7	Family-Patient	0.904
	Chennai	140	7	Government-Patient	0.745
		141	7	Government-Family	0.819
		140	7	Family-Patient	0.787
Family	Montreal	47	7	Government-Patient	0.907
		46	7	Government-Family	0.888
		41	7	Family-Patient	0.879
	Chennai	137	7	Government-Patient	0.749
		137	7	Government-Family	0.722
		137	7	Family-Patient	0.806

All Cronbach’s α values were statistically significant (*p* < 0.05)

**Table 6:** Internal consistency for different language groups from the larger study

Language	Rater	Sample	N	No of items	Pair	Cronbach’s α
English	Patient	Montreal	40	7	Government-Patient	0.885
			41	7	Government-Family	0.916
			41	7	Family-Patient	0.862
		Chennai	33	7	Government-Patient	0.809
			33	7	Government-Family	0.878
			33	7	Family-Patient	0.823
	Family	Montreal	23	7	Government-Patient	0.895
			23	7	Government-Family	0.871
			20	7	Family-Patient	0.89
		Chennai*	5	7	Government-Patient	0.786
			5	7	Government-Family	0.889
			5	7	Family-Patient	0.478
French	Patient	Montreal	27	7	Government-Patient	0.948
			26	7	Government-Family	0.924
			26	7	Family-Patient	0.90
	Family	Montreal	37	7	Government-Patient	0.898
			37	7	Government-Family	0.892
			35	7	Family-Patient	0.893
Tamil	Patient	Chennai	129	7	Government-Patient	0.756
			130	7	Government-Family	0.80
			129	7	Family-Patient	0.799
	Family	Chennai	159	7	Government-Patient	0.75
			159	7	Government-Family	0.746
			159	7	Family-Patient	0.808

All Cronbach’s α values were statistically significant (*p* < 0.05)

*Cronbach’s alpha estimates need to be interpreted with caution as it was based on data from only 5 participants. Only 5 family members responded to the English version of the WRS in Chennai, with the rest opting for the Tamil version

### Frequency distributions of WRS scores

With a few exceptions, scores for each individual item fell in all three ranges (1–3, 4–6 and 7–10) for all stakeholder groups (patients, families, and clinicians) irrespective of site, suggesting that as intended, the scale captured variations in individuals’ views on how responsibility for meeting specific needs should be distributed. There were a small number of cases where the 7–10 portion of the scale was never endorsed (Table 7). In all these instances, these seemed to be reflective of views held by individuals in a particular context or a particular stakeholder group, and were in the expected direction; e.g., patients in Chennai never endorsed scores between 7 and 10 (suggestive of patients being substantially more responsible than families) for addressing housing needs of individuals with mental health problems. Nearly our entire sample and many young persons with and without mental health problems in India live with their families [16, 51–53]. Similarly, clinicians at both sites never endorsed scores between 7 and 10 (suggestive of families being substantially more responsible than the government) on the item about responsibility for covering costs of mental health services, suggesting that they saw this much more as a responsibility to be fulfilled by governments.

**Table 7:** WRS items which were never assigned a score between 7 and 10 at either Time 1 or Time 2 of testing

Stakeholders being apportioned responsibility	Need being rated	Stakeholders who did not assign scores between 7 and 10
Government vs. persons with mental health problems^a^	Financial support	Patients in Chennai
	Coverage for the costs of mental health services	Patients in Chennai
Government vs. families^b^	Coverage for the costs of mental health services	Clinicians, both sites combined; patients in Chennai
Family vs. persons with mental health problems^c^	Provision or support for housing	Patients in Chennai
	Coverage for the costs of mental health services	Patients in Chennai
	Awareness and reduction of stigma surrounding mental health problems	Patients in Chennai; families in Chennai; clinicians at both sites

*vs.* versus

^a^ and ^c^Score of 10 = patients should be more responsible for supporting the specific need

^b^Score of 10 = families should be more responsible for supporting the specific need

### Participant feedback

All participants completed the WRS with minimal support at both sites. Participants in Chennai (N = 10; 5 each in Tamil and English) reported that the WRS was average in terms of ease of completion (M = 4.1 ± 2.02) and comprehension (M = 4.5 ± 1.64), and 8/10 participants found it easy to answer. Feedback from Montreal participants (N = 11; 7 in English, 4 in French) suggested that the WRS was average in terms of ease of completion (M = 5.54 ± 2.94) and comprehension (M = 5.09 ± 2.73); and 6/11 participants found it easy to answer. We did not analyze separately by language given the small sample sizes, but no pattern was discernable *prima facie*.

## Discussion

The aim of this paper was to report on the development and reliability estimates of the WRS, a self-report scale assessing stakeholders’ perceptions of the relative responsibilities of the government, persons with mental health problems, and their families in addressing the support needs of persons with mental health problems. The WRS test–retest reliability was generally found to be excellent to good across the different stakeholder groups (patients, families, and clinicians), settings (Montreal and Chennai), and languages (English, French, and Tamil). Internal consistency estimates were well within the acceptable range and the tool scored in the average range overall in terms of ease of comprehension and completion. In all, the WRS performed adequately with all stakeholder groups and in the three language groups in Chennai and Montreal.

The chief strength of this measure lies in its novelty and its focus on a construct that has generally been ignored in mental healthcare: perceptions of relative responsibility for supporting various needs of individuals with mental health problems. This issue is particularly salient at a time when mental health problems are the subject of increased attention, with substantial discussion in both HIC and LMIC contexts regarding novel infrastructures, funding, and service design. We modelled scale items on an item that has been part of the World Values Survey [32] since 1990 and has proven to be sensitive to capturing differences in individuals’ views about government and individual responsibilities for need provision across countries [54, 55]. Through this tool, we extend this focus on locus of responsibility to mental healthcare. Taken as a whole, the tool allows the assessment of relative (rather than absolute) judgments of responsibility apportioned to governments versus persons with mental health problems, governments versus families, and families versus persons with mental health problems – three key stakeholder groups in mental healthcare, irrespective of geo-cultural context. It also allows for stakeholders to hold varying views about how responsibility should be distributed based on the need area being assessed. This is important, as perceptions of responsibility may vary based on the support need in question [23]. For instance, some stakeholders may generally hold governments more responsible than persons with mental health problems or their families for covering the costs of mental health services but hold persons with mental health problems more responsible for covering the costs of treatment for substance use.

The WRS derives construct and content validity from the construct of perceptions of relative responsibility being well-supported by a critical literature review [23], and the seven need dimensions assessed by the WRS being identified on the basis of previous literature [1, 33–39] and focus groups conducted with patients, families, and clinicians across two different settings in Canada and India [40–42]. Moreover, our findings from the larger Chennai-Montreal study (presented in Part 2) indicate that the WRS can detect hypothesized differences in stakeholders’ perceptions across sites, supporting the validity of this tool. Additional investigations with specified hypotheses can be conducted (e.g., family members who assign higher levels of responsibility to families will be more involved in treatment) to establish predictive validity in future studies.

The WRS is a promising tool with policy, research, and clinical applications. Using tools like the WRS can help ensure that health policy development in specific settings is informed by inputs from all stakeholder groups regarding the priorities and perceived responsibilities of the state versus other actors. Although rarely done to date, such stakeholder consultation can result in greater clarity of roles and responsibilities, strengthened participation of all stakeholders, and citizen-informed health policy [56]. In global health research, the WRS can help measure attitudes about healthcare responsibilities, how they differ across contexts and stakeholder groups, what underpins these differences (e.g., healthcare structures, value systems, etc.), and how these attitudes shape the organization, delivery, and outcomes of health services. While the WRS has more appreciable applications in policymaking and research, clinically, the entire tool or selected items pertaining to particular need areas can be used to foster awareness, dialogue, and understanding among patients, families, and clinicians about roles and responsibilities in the treatment context. Such discussion can promote shared understanding and decision-making, and improve patient-family, patient-provider, and provider-family relationships [57].

### Limitations

Our psychometric testing focused on test–retest and internal consistency reliability, with additional evaluation of the scale's ease of use. Test–retest reliability was higher for clinicians compared to patients and families, which may have resulted from them having found the tool easier to comprehend and complete. However, because the feedback questionnaires were administered only to patients, we are limited in our ability to comment on how families and clinicians perceived the tool. While the majority of patients who completed the feedback questionnaire did not perceive the WRS to be difficult to answer, a minority (7/21 patients) did. As the original intent was for the WRS to be either self-administered or interviewer-administered, we recommend flexible use of the tool, employing an interviewer-administered modality whenever necessary and feasible. However, we do recommend that the modality of administration be consistent across time for each respondent. Future research employing cognitive interviewing [58] and more in-depth user consultation may be helpful in changing instructions for greater clarity and accessibility, and perhaps even in further refining the tool. Notwithstanding the value of such future research, our current efforts are a noteworthy first-ever attempt at measuring views about the relative roles of pertinent stakeholder groups in mental healthcare.

A further limitation of our study is the relatively small size of our standardization sample. Nonetheless, we did recruit from all three stakeholder groups and in two languages (for a total of three languages overall) at both sites. In the case of the clinician group, sample size was limited by the relatively low number of clinicians at each site (most of whom participated in the study). Furthermore, we compensated for the small sample size by at least confirming the internal consistency estimates in larger samples from the main study.

## Conclusion

In conclusion, the WRS is a novel and reliable measure of values related to the central issue of who should provide for which needs in mental healthcare. It can help to operationalize the notion of shared responsibility that, though rarely measured, is widely accepted as an important organizing principle for mental health services delivery and policy. Future research should be conducted in additional LMIC and HIC settings to confirm the reliability, validity and utility of this measure in other contexts.

## Supplementary Information

**Additional file 1. **Whose Responsibility Scale, patient version.

**Additional file 2. **Table: ICC values after excluding data of Chennai participants with very divergent Time 1 and Time 2 scores on the WRS.

## Data Availability

The measure reported on in this paper, Whose Responsibility Scale, is included as Supporting Information. To use or modify the scale, please contact its primary creator at srividya.iyer@mcgill.ca. The datasets generated and/or analysed during the current study are not publicly available due to ethics approval from participants not covering public sharing but are available from the corresponding author on reasonable request.

## Abbreviations

HIC: High-income country
HICs: High-income countries
LMIC: Low- and middle-income country
LMICs: Low- and middle-income countries
WRS: Whose Responsibility Scale
Vs: Versus
