# Supplementary material for: Whose responsibility? Part 1 of 2: A scale to assess how stakeholders apportion responsibilities for addressing the needs of persons with mental health problems
Source: Int J Ment Health Syst. 2022 Jan 10;16:1. doi: 10.1186/s13033-021-00510-x (PMC8744233; doi:10.1186/s13033-021-00510-x)
Supplement: Supplementary file 2 — Additional file 2. Table: ICC values after excluding data of Chennai participants with very divergent Time 1 and Time 2 scores on the WRS. [file 13033_2021_510_MOESM2_ESM.docx]

**Additional File 2: ICC values after excluding data of Chennai participants with very divergent Time 1 and Time 2 scores on the WRS.**

| **SAMPLE** | **DOMAIN** | **Old ICC value** | **New ICC value** |
| --- | --- | --- | --- |
| Patient sample | Govt-Family domain | 0.483 | 0.979 |
| Family sample | Govt-Patient domain | 0.539 | 0.849 |
| Tamil sample | Govt-Patient domain | 0.455 | 0.688 |
